# Association of retinol binding protein 4 and transthyretin with triglyceride levels and insulin resistance in rural thais with high type 2 diabetes risk

**DOI:** 10.1186/s12902-018-0254-2

**Published:** 2018-05-10

**Authors:** Karunee Kwanbunjan, Pornpimol Panprathip, Chanchira Phosat, Noppanath Chumpathat, Naruemon Wechjakwen, Somchai Puduang, Ratchada Auyyuenyong, Ina Henkel, Florian J. Schweigert

**Affiliations:** 1 Department of Tropical Nutrition and Food Science, Faculty of Tropical Medicine, Mahidol University, Bangkok, 10400 Thailand; 2 Department of Nutrition, Faculty of Public Health, Mahidol University, Bangkok, 10400 Thailand; 3 Faculty of Nursing, Huachiew Chalermprakiet University, Samut Prakan, 10540 Thailand; 4 Faculty of Public Health, Nakhonratchasima Rajabhat University, Nakhon Ratchasima, 30000 Thailand; 5 Department of Food Business and Nutrition, Faculty of Agriculture, Ubon Ratchathani Rajabhat University, Ubon Ratchathani, 34000 Thailand; 6 Institute of Nutritional Science, University of Potsdam, 14558 Potsdam, Germany

**Keywords:** RBP4, TTR, HOMA-IR, Hypertriglyceridemia, Type 2 diabetes

## Abstract

**Background:**

Retinol binding protein 4 (RBP4), a protein secreted by adipocytes and bound in plasma to transthyretin (TTR), has been associated with obesity, the early phase of insulin resistance, metabolic syndrome, and type 2 diabetes mellitus. The objective of this study was to elucidate the relationship between RBP4, TTR, triglyceride (TG) and type 2 diabetes risk in rural Thailand.

**Methods:**

We measured the serum RBP4, TTR, glucose, triglyceride and insulin levels, and glucose tolerance of 167 volunteers from Sung Noen District, Nakhon Ratchasima Province, Thailand. Student’s t-test, Pearson’s correlation and logistic regression analysis were used to evaluate the relationships between RBP4, TTR and type 2 diabetes markers.

**Results:**

RBP4 and TTR levels, as well as homeostatic model assessment of insulin resistance (HOMA-IR) values, were significantly elevated among subjects with high triglyceride levels (*p* < 0.01, *p* < 0.05, p < 0.05, respectively). Triglyceride levels correlated with RBP4 (*r* = 0.34, *p* < 0.001) and TTR (*r* = 0.26, *p* < 0.01) levels, as well as HOMA-IR values (*r* = 0.16, *p* < 0.05). After adjustment for age and gender, the risk of hypertriglyceridemia was 3.7 times greater (95% CI =1.42–9.73, *p* = 0.008) in the highest RBP4 tertile as compared to the lowest tertile. Similarly, the highest TTR and HOMA-IR tertiles had greater risk of hypertriglyceridemia at 3.5 (95% CI = 1.30–9.20, *p* = 0.01) and 3.6 (95% CI = 1.33–9.58, *p* = 0.01) times higher than the respective lowest tertiles. The correlation between TTR and blood glucose was statistically significant (*r* = 0.18, *p* < 0.05), but not found this relationship in RBP4.

**Conclusions:**

The associations of RBP4 and TTR with hypertriglyceridemia and insulin resistance may have important implications for the risk of heart disease and stroke.

## Background

Retinol binding protein (RBP4) is an adipokine that may be linked to type 2 diabetes (T2DM), which leads to cardiovascular disease (CVD). Several studies have reported elevated plasma RBP4 in T2DM subjects with obesity [1], impaired glucose tolerance [2–4], and T2DM with nephropathy [5]. RBP4 has also been found to affect the insulin signaling cascade, leading to insulin resistance. This adipokine is also associated with CVD, a complication induced by diabetes. While it has been shown that elevated serum RBP4 levels manifest in the development of systemic insulin resistance in rats [6], evidence for an effect of RBP4 on obesity and insulin resistance in humans is controversial. Studies have reported early states of T2DM with both increased and decreased levels of RBP4 [7–10]. Transthyretin (TTR), a transport protein, carries RBP4 and the thyroid hormone, thyroxin (T4), through the blood [11]. Recently, a human study found that circulatory RBP4 and TTR is associated with glucose intolerance and obesity, and T2DM and RBP4 is associated with insulin resistance [12].

Elevated plasma triglyceride (TG) concentration is a common biochemical finding associated with insulin resistance and is a valuable clinical marker of the metabolic syndrome. Observational and meta-analytic studies have shown relationships between increased cardiovascular risk and hypertriglyceridemia [13–15]. Numerous and complex cases of dyslipidemia, hypertension, hypercoagulability, and atherosclerosis are linked with insulin resistance. The inability of insulin-resistant fat cells to store TG is very likely the initial step in the development of the dyslipidemia. Based on in-vitro studies, short-term increases in insulin levels are associated with increased TG synthesis [16].

Metabolic syndrome consists of a clustering of several metabolic risk factors in an individual and is a major component of atherogenic dislipidemia, increased blood pressure, elevated glucose, and a prothrombotic state [17]. T2DM is a major public health problem in Thailand, with over 4 million cases of diabetes reported in 2015 [18, 19]. CVD has been the leading cause of death for over a decade and diabetes is likely to be an important factor in the vascular disease burden [20–22]. The objective of the present study was to investigate whether RBP4 and TTR are associated with insulin resistance, prediabetes and triglyceride levels in rural Thais with high T2DM risk**.**

## Methods

### Subjects

This cross-sectional study involved 167 participants aged between 35 and 66 years, from Sung Noen District, Nakhon Ratchasima Province, Thailand. Subjects were randomized and free of baseline T2DM and other any chronic diseases. Those pregnant, within a 6-month lactation period, or those regularly taking any medicine or having any infections, were excluded from the study. Informed consent was obtained from the subjects after explaining the study procedures in detail. The study protocol was approved by the Ethics Committee of the Faculty of Tropical Medicine (TMEC 13–073), Mahidol University.

### Anthropometric assessment

Each subject was weighed (in kilograms) and measured for height (in meters) to evaluate individual body mass index (BMI, kg/m^2^). Waist circumference (WC) and hip circumference (HC) were measured and used to determine waist-to-hip ratio (WHR). A body composition monitor (model HBF-375, Omron Healthcare, Kyoto, Japan) was used to determine each subject’s body composition, including percentage of body fat (BF), visceral fat (VF), and muscle.

### Measurement of laboratory parameters

Blood samples were taken from the antecubital veins after a fasting period of at least 12 h, and were used to assess fasting blood glucose (FBG), glycohemoglobin (HbA_1c_), fasting insulin, total cholesterol (TC), HDL-c and TG. Another blood sample was taken 2 h after a 75 g oral glucose load to determine glucose tolerance. Blood samples were centrifuged and blood serum was immediately frozen at 80 °C until analysis. FBG, HbA_1c_, 2-h blood glucose (2hBG), and TG were analyzed using a Cobas 6000 analyzer (Roche Diagnostics Ltd., Basel, Switzerland). A human insulin ELISA kit (EMD Millipore, Billerica, MA, USA) was used to measure fasting insulin levels. Insulin resistance was evaluated by the homeostatic model assessment of insulin resistance (HOMA-IR) i.e. HOMA-IR = [Fasting insulin (μIU/mL) × Fasting glucose (mmol/L)] / 22.5.

TC and HDL-c were determined by enzymatic assay (Thermo Fisher Scientific Inc., Waltham, Massachusetts, USA). Levels of TC, HDL-c, and TG were then used to calculate low density lipoprotein cholesterol (LDL-c) using the Friedewald equation i.e. LDL-c = TC – (HDL-c + TG / 5). Serum RBP4 and TTR levels were assessed by non-commercial enzyme-linked immunosorbent assays using polyclonal rabbit anti-human antibodies (DakoCytomation, Hamburg, Germany), as previously described [23]. Both assays were calibrated using the standards obtained from human blood (N Protein Standard SL; Dade Behring, Marburg, Germany).

### Dietary assessment

A validated semi-food frequency questionnaire (semi-FFQ) containing checklists of various food and beverages, food portion sizes, and consumption frequencies, was used to estimate dietary intake. Energy, protein, carbohydrate and fat intake were calculated using NutriSurvey software (version 2007; SEAMEO-TROPMED RCCN, University of Indonesia).

### Statistical analysis

Statistical analysis was conducted using SPSS (version 15.0; SPSS, Chicago, IL, USA), with results expressed as mean and standard deviation. A Student’s t-test was performed to compare the means between the two groups. Correlations among variables were calculated using Pearson’s correlation with the associations estimated using odd ratios (OR) and 95% confidence intervals (CI) obtained from logistic regression. *P* values < 0.05 were considered statistically significant.

## Results

### Biometric and biochemical characteristics of the study groups

The study participants were divided into two groups according to TG level using a 150 mg/dl cut-off point [24]. The group with normal TG levels (TG < 150 mg/dl) contained 110 subjects, with the remaining 57 subjects positive for hypertriglyceridemia (TG ≥ 150 mg/dl). A comparison of age, BMI, WC, HC, WHR, BF, VF, and muscle between normal and high TG subjects is shown in Table 1. Normal and high TG subjects were in the same age range. Body size and composition, measured by BMI, HC, WHR, BF, and muscle, showed no significant differences between normal and high TG groups. Conversely, the central obesity indicators WC and VF were significantly elevated in the high TG group (*p* < 0.05). Likewise, the high TG group had higher diastolic blood pressure readings than the normal TG group (*p* = 0.03), though there were no significant differences between groups for systolic blood pressure, FBG, 2hBG, and HbA_1c_. Regarding lipid profiles, there were no significant differences in TC, HDL-c, or LDL-c between the normal and high TG groups. The results from the semi-FFQ showed that the normal- and high TG groups had similar dietary intake patterns. The average energy intake of the normal TG group was 2404.82 kcal/day, and of the high TG group 2382.24 kcal/day, with carbohydrates constituting the highest proportion by weight (Table 2).

**Table 1:** Characteristic of the study group

Variables	Normal TG group (*n* = 110)	High TG group (*n* = 57)	*P*-value
Gender (male/female)	32/79	15/42	0.80*
Age (year)	46.27 ± 5.92	47.26 ± 5.94	0.31
Hip circumference (cm)	92.35 ± 11.43	94.21 ± 8.34	0.28
Waist-hip ratio	0.89 ± 0.09	0.91 ± 0.06	0.30
Body fat (%)	29.86 ± 7.96	30.59 ± 6.81	0.55
Visceral fat (%)	8.70 ± 5.09	10.48 ± 5.54	0.04
Muscle (%)	26.11 ± 4.05	25.75 ± 3.62	0.57
Systolic blood pressure (mmHg)	122.75 ± 18.58	123.88 ± 20.94	0.72
Diastolic blood pressure (mmHg)	72.55 ± 10.45	76.98 ± 13.53	0.03
Fasting blood glucose (mg/dL)	94.22 ± 13.43	96.18 ± 23.55	0.49
2hBG (mg/dL)	122.67 ± 49.62	137.46 ± 70.90	0.12
HbA_1c_ (%)	5.43 ± 0.67	5.54 ± 1.04	0.43
Total cholesterol (mg/dL)	200.23 ± 58.94	212.14 ± 63.17	0.23
LDL-C (mg/dL)	149.58 ± 61.17	165.63 ± 63.42	0.11
HDL-C (mg/dL)	50.65 ± 14.89	46.51 ± 15.29	0.09

Data are presented as means ±standard deviation unless otherwise specified

*P*-values were calculated by Student’s t-test

*P-value for gender were calculated by Chi-square test

**Table 2:** Dietary intake of the study group

Variables	Normal group (*n* = 110)	High TG group (*n* = 57)	*P*-value
Energy intake per day (kcal)	2404.82 ± 681.95	2382.24 ± 815.35	0.87
Protein intake per day (g)	75.76 ± 37.85	69.78 ± 32.82	0.31
Fat intake per day (g)	65.94 ± 39.16	60.58 ± 42.80	0.42
Carbohydrate intake per day (g)	369.59 ± 119.48	377.80 ± 120.19	0.68

Data are presented as means ±standard deviation unless otherwise specified

*P*-values were calculated by Student’s t-test

### Increased RBP4 and TTR levels and HOMA-IR values in the high TG group

**Fig. 1:**
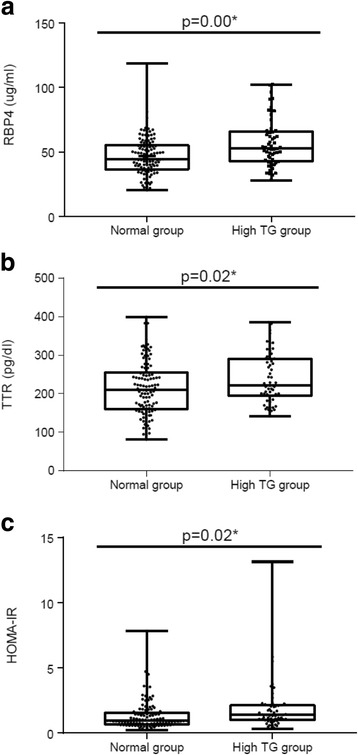
Comparison of RBP 4 (**a**), TTR levels (**b**) and HOMA-IR (**c**) value between normal- and high TG groups. *P*-values were calculated by Student’s t-test. RBP4: retinal binding protein 4, TTR: transthyretin, HOMA-IR: homeostatic model assessment of insulin resistance

Serum RBP4 and TTR were significantly elevated in the high TG group (*p* < 0.05) (Fig. 1a, b). HOMA-IR values corresponded to the levels of RBP4 and TTR, with the high TG group showing higher HOMA-IR values than the normal TG group (*p* = 0.02) (Fig. 1c). Significant positive correlations were found between serum TG and RBP4 (*r* = 0.34, *p* = 0.000), TTR (*r* = 0.26, *p* = 0.001), and HOMA-IR (*r* = 0.16, *p* = 0.039).

The risk of hypertriglyceridemia was evaluated by multivariate analysis (Table 3). Fasting RBP4 and TTR levels were divided into tertiles, with the lowest tertile being the reference tertile. The results showed that after adjustment for age and gender, individuals with RBP4 levels in the highest tertile had 3.7 times higher risk of developing hypertriglyceridemia than those in the lowest tertile (OR = 3.71, 95% CI = 1.42–9.73). Similarly, subjects in the highest TTR and HOMA-IR tertiles had 3.5 times (OR = 3.46, 95% CI = 1.30–9.20) and 3.6 times (OR = 3.58, 95% CI = 1.33–9.58) higher risk of developing hypertriglyceridemia, respectively, than those in the lowest tertiles.

**Table 3:** Association between RBP4, TTR, HOMA-IR and risk of hypertriglyceridemia

Variables	Subjects		Odd ratio^a^	95% CI	*P*-value
	Control	Case			
RBP4 (μg/mL)					
≤ 39.27	33	9	1		
39.28–58.81	57	27	1.67	0.70–4.01	0.25
≥ 58.82	20	21	3.71	1.42–9.73	0.008
	110	57			
TTR (pg/mL)					
≤ 169.26	33	9	1		
169.27–272.51	55	29	2.01	0.84–4.81	0.12
≥ 272.52	22	19	3.46	1.30–9.20	0.01
	110	57			
HOMA-IR					
≤ 0.75	36	9	1		
0.76–1.80	52	29	2.23	0.92–5.39	0.08
≥ 1.81	22	19	3.58	1.33–9.58	0.01
	110	57			

^a^Odd ratios were adjusted by age and gender

95% CI and *P*-values were calculated by logistics regression model

### Association of RBP4, TTR, and HOMA-IR with serum glucose

Pearson correlation coefficients were used to assess the relationship between blood glucose and study parameters. Blood glucose showed a non-significant positive correlation with most of the study parameters, including WHR (*r* = 0.031), systolic (*r* = 0.194) and diastolic (*r* = 0.112) blood pressure, VF (*r* = 0.036), muscle (*r* = 0.098), TC (*r* = 0.025), LDL-c (*r* = 0.001), and HDL-c (*r* = 0.095). To investigate T2DM risk, the study subjects were divided into two groups based on FBG, i.e. normal T2DM risk group (FBG < 100 mg/dl) and high T2DM risk group (FBG≥100 mg/dl). The high T2DM risk group showed increased TTR levels (*p* = 0.02) and HOMA-IR values (*p* = 0.03), as well as decreased RBP4 levels (*p* = 0.04) than the normal T2DM risk group (Table 4).

**Table 4:** Comparison of RBP4, TTR, and HOMA-IR between normal group and T2DM risk group base on fasting blood glucose levels

Variables	FBG< 100 mg/dL (*n* = 122)	FBG ≥ 100 mg/dL (*n* = 45)	*P*- value
RBP 4 (μg/mL)	51.87 ± 17.41	45.95 ± 13.74	0.04
TTR (pg/mL)	215.36 ± 67.98	242.65 ± 66.49	0.02
HOMA-IR	1.36 ± 1.42	1.90 ± 1.32	0.03

Data are presented as means ±standard deviation unless otherwise specified

*P*-values were calculated by Student’s t-test

### Correlation between RBP4, TTR, and HOMA-IR with metabolic syndrome parameters

Pearson correlation coefficients were used to assess the relationship between RBP4, TTR, and HOMA-IR, with metabolic syndrome parameters. There were many statistically significant correlations between RBP4, TTR, and HOMA-IR, with metabolic syndrome parameters including RBP4 correlated with TG (*r* = 0.344) and HDL-c (*r* = 0.259); TTR correlated with FBG (*r* = 0.182), TG (*r* = 0.260), and HDL-c (*r* = 0.168) was also significant; HOMA-IR correlated with FBG (*r* = 0.216), TG (*r* = 0.160), and HDL-c (*r* = 0.186). RBP4 showed a negative correlation with FBG (*r* = − 0.057) and a positive correlation with HOMA-IR (*r* = 0.134), but without statistical significance. TTR was also found to be non-statistically significant with HOMA-IR (*r* = − 0.046). However, the correlation between TTR and FBG was statistically significant (r = 0.182).

## Discussion

The subjects with hypertriglyceridemia in this study presented a higher risk of metabolic syndrome due to their above normal TG levels and abdominal obesity indicator values (WC and VF). These subjects may also have increased insulin resistance, since they showed increased HOMA-IR values. Many other investigators have reported that hypertriglyceridemia is closely related to an insulin resistant state. Patients with primary hypertriglyceridemia have increased non-esterified fatty acid turnover rates and secretion by adipose tissue, supplying an excess of fatty acids to the liver for synthesis of TG. This increase suggests that patients with hypertriglyceridemia have insulin resistance at the level of the adipose tissues [25–27]. Björntorp suggests that abdominal obesity is closely linked to insulin resistance, because the mobilized TG in visceral fat creates large amounts of free fatty acid in the portal vein, which can affect liver function and lead to hypertriglyceridemia [28].

There is evidence to suggest that RBP4 and TTR play a role in the development of insulin resistance [1, 12, 29] and metabolic syndrome [30–32]. RBP4 was found to interrupt the insulin signaling cascade causing insulin resistance. In addition, relationships have been found between RBP4 and diabetes complications, such as atherosclerosis and CVD [6]. Several studies have investigated the association of RBP4 with T2DM risk, however the results are conflicting. In a cross-sectional study by Comucci et al., Brazilian subjects with T2DM showed lower RBP4 levels than the control group, though RBP4 was not related to T2DM [33]. TTR functions as a carrier for RBP4, and since hepatic secretion of TTR is affected by dietary protein and energy intake, TTR is also used as a biomarker for assessing nutritional status [34]. TTR levels were reported to be elevated in T2DM subjects and to correlate positively with TG level [30]. Pandey et al. have shown higher circulatory levels of RBP4 and TTR in T2DM subjects, as well as a significant association between T2DM and RBP4 (OR = 1.11, 95% CI: 1.01–1.21) and TTR (OR = 1.34, 95% CI: 1.17–1.55) after adjusting for confounding factors [12]. In the present study, subjects with FBG ≥100 mg/dl had significantly lower serum levels of RBP4 and higher levels of TTR than those with FBG < 100 mg/dl. Moreover, a significant correlation was found between FBG and TTR levels (*p* = 0.018), although the results for a correlation between FBG and RBP4 levels were inconclusive. Moreover, a relationship between RBP4, TTR and metabolic syndrome components was found in this study. Classified by TG level, subjects with high TG showed increased RBP4 and TTR levels, as well as increased HOMA-IR values. In addition, the risk of hypertriglyceridemia was approximately 4-fold greater in subjects with high serum RBP4 and TTR levels and HOMA-IR values. An association between increased levels of both RBP4 and TTR and an elevated risk of insulin resistance (the precursor state to T2DM) and CVD (the macrovascular complication of diabetes) has been reported in several studies [1, 2, 35]. A positive correlation has also been found between plasma RBP4 and TG levels in subjects with T2DM [7, 35, 36]. Hypertriglyceridemia has been independently associated with an elevated risk of CVD [37, 38]. However, while associations between plasma RBP4 levels, TG levels and insulin resistance have been shown in many human studies, while other studies report an insulin resistance-independent association between plasma RBP4 and TG levels [7, 9]. Nevertheless, the results of the present study suggest associations between RBP4, TTR, TG, and insulin resistance. In particular, our results indicate that TTR may play a role in the pathophysiology of diabetic hypertriglyceridemia.

## Conclusions

The relationships between RBP4 and TTR levels and FBG abnormalities in this study remain ambiguous. Conversely, our results show a significant association between serum TG levels and insulin resistance. TTR was associated with prediabetes and TG level, whereas RBP4 was only associated with TG. Our findings indicated that RBP4 and TTR are related to hypertriglyceridemia and insulin resistance; therefore, these markers may involve the risk of heart disease and stroke. However, the cross-sectional nature of this study means that these associations reflect a single time point only. A cohort study is therefore required to confirm the associations of these markers with blood glucose and serum TG levels.

## Abbreviations

2hBG: 2-h blood glucose
95% CI: 95% confidence intervals
BF: Body fat
BMI: Body mass index
CVD: Cardiovascular disease
FBG: Fasting blood glucose
HbA1c: Glycated hemoglobin
HC: Hip circumference
HDL-c: High density lipoprotein cholesterol
HOMA-IR: The homeostatic model assessment of insulin resistance
LDL-c: Low density lipoprotein cholesterol
*p*: *p*-value
r: Regression coefficient
RBP4: Retinol binding protein 4
Semi-FFQ: Semi-quantitative food frequency questionnaire
T2DM: Type 2 diabetes mellitus
TC: Total cholesterol
TG: Triglyceride
TTR: Transthyretin
VF: Visceral fat
WC: Waist circumference
WHR: Waist to hip ratio
